# Integrated Analysis of the Functions and Prognostic Values of RNA-Binding Proteins in Colorectal Cancer

**DOI:** 10.3389/fcell.2020.595605

**Published:** 2020-11-05

**Authors:** Ya Wang, Yuqiao Chen, Shuai Xiao, Kai Fu

**Affiliations:** ^1^Institute of Molecular Precision Medicine and Hunan Key Laboratory of Molecular Precision Medicine, Xiangya Hospital, Central South University, Changsha, China; ^2^Department of General Surgery, Xiangya Hospital, Central South University, Changsha, China; ^3^Department of Gastrointestinal Surgery and Institute of Clinical Medicine, The First Affiliated Hospital, University of South China, Hengyang, China; ^4^Center for Medical Genetics & Hunan Key Laboratory of Medical Genetics, School of Life Sciences, Central South University, Changsha, China; ^5^Hunan Key Laboratory of Animal Models for Human Diseases, Central South University, Changsha, China

**Keywords:** colorectal cancer, RNA-binding protein, prognostic signature, overall survival, regulatory network

## Abstract

Colorectal cancer (CRC) is one of the most common malignant tumors. Selecting effective treatment for CRC patients, especially in the early stages, remains a challenge because of the lack of adequate biomarkers. Recent evidence suggests that RNA-binding proteins (RBPs) play a vital role in development and progression of carcinogenesis. However, their mechanisms in cancer progression are still limited. The role of RBPs in CRC has been poorly understood. There were 1,542 reported RBPs analyzed between CRC tissues and normal tissues using the Wilcoxon test to identify differentially expressed RBPs (DE RBPs). Then, the potential functions and the prognostic value of these DE RBPs were explored through systematic bioinformatics analysis. There were 177 DE RBPs identified between CRC tissues and normal tissues. A protein–protein interaction network was constructed based on DE RBPs, and critical modules were screened. A regulatory network between prognostic DE RBPs and differentially expressed transcription factors was constructed. Besides, a risk signature was built based on prognostic DE RBPs, which is able to predict overall survival of CRC patients with high accuracy. In conclusion, the results provided a comprehensive understanding of the functions of RBPs in CRC, as well as an RBP-related prognostic signature.

## Introduction

Colorectal cancer (CRC) is one of the most common malignant tumors and the second cause of tumor-related mortality worldwide (Arnold et al., 2017; Bray et al., 2018). By 2030, the global burden of CRC is expected to increase by 60%, with 2.2 million new cases and 1.1 million deaths (Douaiher et al., 2017). The 5-year survival for CRC patients with local tumor is 90.3% and is 70.4% for patients with locally advanced disease, which declines to 12.5% for patients with metastatic disease (DeSantis et al., 2014). Surgery is the most common treatment for early CRC and locally advanced CRC (Hudson et al., 2013; Angenete, 2019). However, half of the patients will suffer a recurrence within 3 years after surgery (Aghili et al., 2010). Despite some advances in the diagnosis and treatment of CRC over the past few decades, the 5-year overall survival (OS) is only 50–65% (DeSantis et al., 2014). Selecting effective treatment for CRC patients, especially in the early stages, remains a challenge because of the lack of adequate biomarkers. Therefore, further understanding of the pathogenesis of CRC will help us to develop effective means for diagnostic and treatment.

It is widely believed that the development of cancer is partly determined by abnormal transcription events and signaling pathways. Emerging evidence shows that RNA-binding proteins (RBPs) could regulate cell proliferation, differentiation, invasion, metastasis, apoptosis, and angiogenesis and thus play a vital role in the initiation and progression of cancer (Wurth, 2012). RBPs regulate a variety of RNA biogenesis, including RNA splicing, polyadenylation, nucleocytoplasmic transport, mRNA translation, and RNA degradation (Lu et al., 2006; Saunus et al., 2008; Shen and Pili, 2008). So far, several RBPs have been found to be involved in cancer progression. RBPs regulate posttranscriptional genes at different levels of mRNA metabolism, including alternative splicing, localization, stability of mRNA, and so on. For example, Sam68, belonging to STAR (signal transduction and activation of RNA) family of RBPs (Bielli et al., 2011; Fu et al., 2019), regulates alternative splicing of cancer-related mRNA, such as CD44 (Matter et al., 2002), cyclin D1 (Paronetto et al., 2010), and Bcl-x (Paronetto et al., 2007). Cap-binding protein eIF4E (eukaryotic initiation factor 4E) is a crucial regulator of translation initiation (Topisirovic et al., 2011). eIF4E has been reported to be overexpressed in different cancers (Hsieh and Ruggero, 2010). However, overexpression of eIF4E does not lead to an overall increase of protein synthesis but enhances the translation of several mRNAs encoding mainly pro-oncogenic proteins (Silvera et al., 2010). mRNAs regulated by eIF4E overexpression include those involving in cell cycle (cyclin D1, CDK2, c-myc, Bcl-2, survivin) or angiogenesis (vascular endothelial growth factor, fibroblast growth factor 2, platelet-derived growth factor) or invasion (matrix metalloproteinase 9) (Mamane et al., 2004; Hsieh and Ruggero, 2010; Silvera et al., 2010). HuR is a member of the ELAV-like family of RBPs. It contains three RNA-recognition motifs by which it binds specific mRNAs to affect their stability and translation. HuR can stabilize the mRNAs coding for cyclins involved in cell cycle progression to promote the proliferation of cancer cells (Wang et al., 2000; Lal et al., 2004; Guo and Hartley, 2006). HuR also stabilizes the mRNAs coding antiapoptotic proteins such as Bcl-2, SIRT1, and p21 (Abdelmohsen et al., 2007; Ishimaru et al., 2009; Cho et al., 2010). Overexpression of HuR has been observed in multiple cancers, including CRC (Lopez, de Silanes et al., 2003; Denkert et al., 2006). HuR has been reported to augment the stability and translation of COX-2 mRNA in CRC (Dixon et al., 2001; Subbaramaiah et al., 2003). Besides, several other RBPs have been found to be abnormally expressed in CRC, such as RBM3 (Sureban et al., 2008), CUGBP2 (Ramalingam et al., 2008), Musashi-1 (Kudinov et al., 2017), and tristetraprolin (Young et al., 2009). However, the RBPs and their mechanisms in cancer progression remain to be explored. Benefiting from high-throughput screening technology, more than 1,500 RBPs have been identified (Gerstberger et al., 2014). A systematic functional study of RBPs in CRC will not only contribute to our deeper understanding of RBPs, but also provide new ideas for the pathogenesis of CRC. Therefore, an integrated bioinformatics analysis of RBPs in CRC was performed. Abnormally expressed RBPs between CRC tissues and normal tissues from The Cancer Genome Atlas (TCGA) dataset were identified, and then a systematic functional analysis was performed on these RBPs. In addition, a prognostic signature related to RBPs was constructed to predict the survival of CRC patients.

## Materials and Methods

### Data Collection and Identification of Differentially Expressed RBPs

Colorectal cancer samples containing genetic information and clinical information from TCGA-COAD were downloaded. RBP lists were obtained as described (Gerstberger et al., 2014). After data normalization, differentially expressed RBPs (DE RBPs) between normal tissues and tumor tissues were identified through the Wilcoxon test. RBPs with |log_2_FC (fold change) | ≥1 and adjusted *p* < 0.05 were deemed DE RBPs.

### Gene Ontology and Kyoto Encyclopedia of Genes and Genomes Functional Enrichment Analyses

Gene Ontology (GO) and Kyoto Encyclopedia of Genes and Genomes (KEGG) enrichment analyses for DE RBPs were performed using an R package “clusterProfiler.” GO annotation based on three categories, including biological processes (BPs), cellular compartments (CCs), and molecular functions (MFs). Terms in GO and KEGG with a false discovery rate <0.05 were considered significantly enriched and were visualized by R package “ggplot2.”

### Protein–Protein Interaction Network Construction and Module Screening

Protein–protein interaction (PPI) network of DE RBPs was constructed using STRING database,^1^ and an interaction with a combined score >0.4 was considered as statistically significant. Cytoscape, an open-source bioinformatics software platform, was used to visualize molecular interaction networks. The plug-in Molecular Complex Detection (MCODE) in Cytoscape software was used to cluster a given network based on the topology to find densely connected regions. The PPI networks were drawn with Cytoscape, and the most significant modules in the PPI networks were identified through MCODE with the default criteria, including MCODE score >5, degree cutoff value = 2, node score cutoff value = 0.2, maximum depth = 100, and *k-*score = 2.

### Construction and Validation of a Prognostic Signature Based on DE RBPs

The CRC patients were randomly divided into a training set and a testing set. The training set was used to construct an RBP-related prognostic signature, and the testing set was used to validate its prognostic capability. To explore the putative DE RBPs related to the prognosis of CRC patients, a univariate Cox proportional hazards regression analysis was performed to explore the putative DE RBPs related to OS of CRC patients. The DE RBP with *p* < 0.05 was considered as a prognostic RBP. Then prognostic RBPs were used to construct the risk model for predicting the prognosis for CRC patients based on a multivariate Cox proportional hazards regression analysis. In this risk model, the risk score of each sample was calculated according to the following formula: risk score = expression of RBP 1 × coefficient 1 + expression of RBP 2 × coefficient 2 + expression of RBP n × coefficient *n*. The median risk score was used as the cutoff value. Patients were then divided into high- and low-risk groups according to the cutoff value. The Kaplan–Meier survival curves were carried out using “survival” R package based on high- and low-risk groups according to the risk model. To validate the prognostic capability of the risk model, the time-dependent receiver operating characteristic (ROC) analyses were performed with “survivalROC” R package.

### Association Between the Risk Signature Related to RBPs and Clinicopathological Factors

To assess whether the prognosis signature we constructed is independent of other clinical factors, a univariate Cox proportional hazards regression and a multivariate Cox regression analysis were performed on the RBP-related signature together with gender, age, and stage.

### The Regulatory Network Between Prognostic DE RBPs and DE Transcription Factors

To explore the potential molecular mechanisms of prognostic DE RBPs, the regulatory network between prognostic DE RBPs and differentially expressed transcription factor (DE TFs) was constructed. TFs are important molecules that directly regulate gene expression. Hence, exploring DE TFs, which have the potential ability in regulating the prognostic DE RBPs, would help to understand the MFs of the prognostic DE RBPs. The Cristrome Cancer database^2^ is a valuable resource for experimental and computational cancer biology research and contains a total of 318 TFs related to cancer (Mei et al., 2017). DE TFs between CRC tissues and normal tissues were identified through the Wilcoxon test. TFs with | log_2_FC | ≥1 and adjusted *p* < 0.05 were deemed DE TFs.

## Results

### Identification of DE RBPs in CRC Patients

**FIGURE 1:**
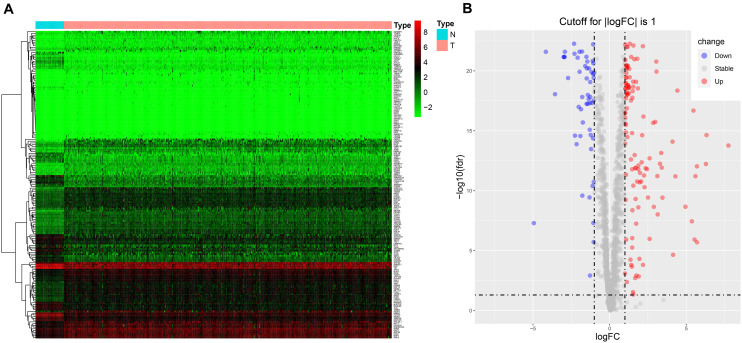
The expression of DE RBPs in CRC based on data from TCGA. **(A)** Heatmap analysis of the differentially expressed RBPs between normal tissues and tumor tissues. Each column represents a tissue sample, and each row represents a differentially expressed RBP. Red indicates high expression, and green indicates low expression. N, normal; T, tumor. **(B)** Volcano plot of differentially expressed RBPs. The horizontal line is for adj *p* = 0.05, and the vertical lines are for | log_2_FC| = 1.

The workflow of the study is illustrated in Supplementary Figure 1. RNA sequencing data for CRC and corresponding clinical information were downloaded from TCGA database. There were 1,542 reported RBPs analyzed (Gerstberger et al., 2014). After data normalization, DE RBPs were detected between 47 normal tissues and 473 tumor tissues with an adjusted *p* < 0.05 and |log_2_FC| ≥ 1 as the thresholds. A total of 177 DE RBPs were obtained (Figure 1A and Supplementary Table 1), including 123 upregulated RBPs and 54 downregulated RBPs in tumor tissues (Figure 1B).

### Functional Enrichment Analysis of the Differentially Expressed RBPs

**FIGURE 2:**
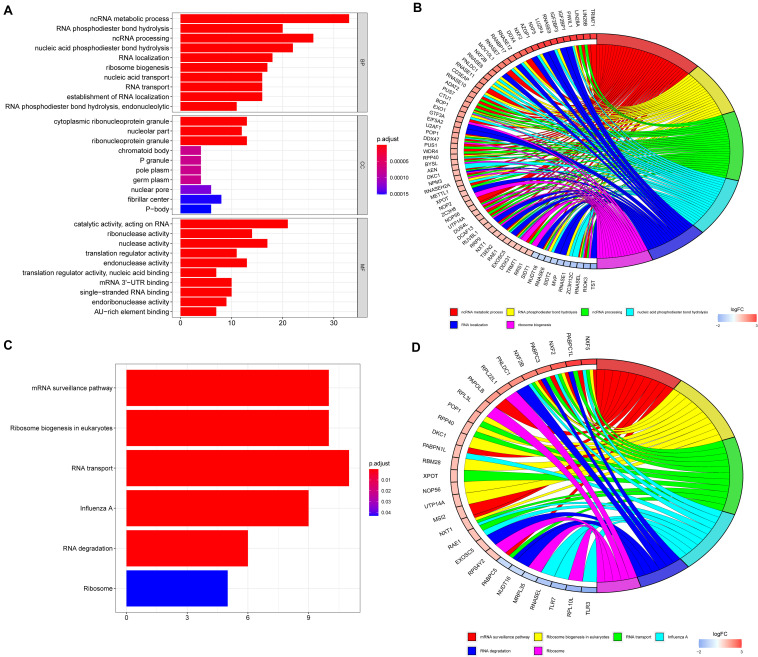
GO and KEGG enrichment analyses of differentially expressed RBPs. **(A)** GO enrichment analysis including biological processes (BP), cellular compartments (CCs), and molecular functions (MFs). **(B)** The top six most significant GO enrichment terms. **(C)** KEGG enrichment analysis. **(D)** The top six most significant KEGG enrichment terms.

To explore the potential functions of the DE RBPs, GO, and KEGG enrichment analyses were performed. For BP enrichment, DE RBPs were mostly enriched in ncRNA (non-coding RNAs) metabolic process, RNA phosphodiester bond hydrolysis, and ncRNA processing (Figure 2A). For CC enrichment, DE RBPs were mainly involved in cytoplasmic ribonucleoprotein granule, nucleolar part, and ribonucleoprotein granule (Figure 2A). For MF enrichment, RBPs are mainly enriched in catalytic activity, acting on RNA, ribonuclease activity, and nuclease activity (Figure 2A). The top five most significant GO enrichment terms are shown in Figure 2B. KEGG enrichment analysis showed DE RBPs were mainly involved in mRNA surveillance pathway, ribosome biogenesis in eukaryotes, and RNA transport (Figures 2C,D).

### PPI Network Construction and Critical Module Screening

**FIGURE 3:**
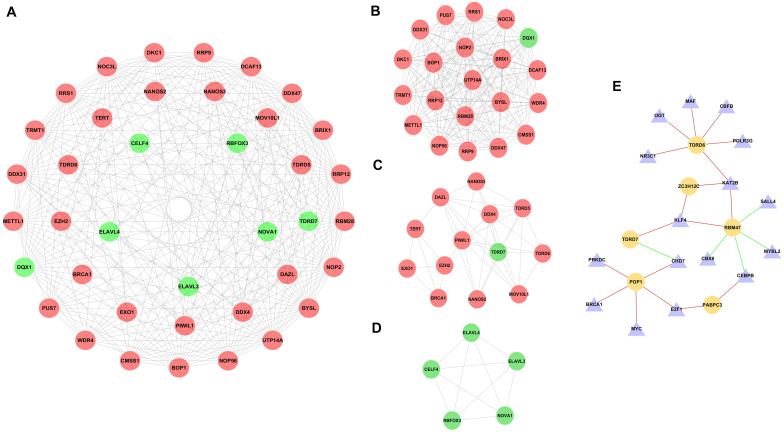
PPI network of differentially expressed RBPs (DE RBPs) and regulatory network based on DE transcription factors and prognostic DE RBPs. **(A)** PPI network including three modules. The three circles from the inside out represent three modules. **(B)** Module 1 in PPI network. **(C)** Module 2 in PPI network. **(D)** Module 3 in PPI network. **(E)** Regulatory network between DE TFs and prognostic DE RBPs. The triangle represents TF, and the cycle represents prognostic DE RBP. The red line represents positive regulation, whereas the green line represents negative regulation.

To further explore the potential functions of DE RBPs, a PPI network was constructed using STRING database and Cytoscape software. The PPI network of DE RBPs was downloaded from STRING database (Supplementary Figure 2A) and visualized with Cytoscape (Supplementary Figure 2B). Then, the network was further analyzed for critical modules by the plug-in MCODE in Cytoscape (Figure 3A), and the top three significant modules were obtained. Module 1 included 21 RBPs, all of which were upregulated RBPs except for DQX1 (Figure 3B). Module 2 included 13 RBPs, all of which were upregulated RBPs except for TDRD7 (Figure 3C). Module 3 included five downregulated RBPs (Figure 3D).

### Identification of Prognosis-Related DE RBPs

To explore the putative DE RBPs related to prognosis for CRC patients, a univariate Cox proportional hazards regression analysis was performed to identify survival-related DE RBPs. There were 13 DE RBPs significantly correlated with OS (*p* < 0.05) (Table 1).

**TABLE 1:** Univariate Cox proportional hazards regression analysis of DE RBPs.

DE RBP	HR	HR.95L	HR.95H	*p-*Value
TDRD6	0.146237	0.031935	0.669646	**0.013267**
POP1	0.547387	0.32844	0.912291	**0.020767**
TDRD7	0.369575	0.198223	0.689049	**0.001738**
LUZP4	9.613842	3.401484	27.17225	**1.96E-05**
PPARGC1B	0.369954	0.164463	0.832203	**0.016215**
PPARGC1A	0.465469	0.28667	0.755786	**0.001987**
PABPC3	0.465077	0.247277	0.874714	**0.017535**
LRRFIP2	0.327354	0.128233	0.83567	**0.019523**
ZC3H12C	0.578625	0.367349	0.911415	**0.01827**
RBM47	0.549608	0.307602	0.982011	**0.043253**
CELF4	27.334	6.51501	114.6809	**6.14E-06**
PNLDC1	1.566855	1.103357	2.225058	**0.012086**
AFF3	4.557023	1.086579	19.11179	**0.038127**

*Bold values significantly changed (*p* < 0.05).*

### The Regulatory Network Between Prognostic DE RBPs and DE TFs

Three hundred eighteen TFs related to cancer were obtained from The Cristrome Cancer database (see text footnote 2). Then, differentially expressed TFs between CRC tumor tissues and normal tissues were analyzed. A total of 77 differentially expressed TFs were obtained with a threshold that adjusted *p* < 0.05 and | log_2_FC| ≥ 1 (Supplementary Table 2). Then, a regulatory network based on these 77 DE TFs and 13 prognostic DE RBPs was constructed (Figure 3E). The coexpressed genes were identified by calculating the Pearson correlation between DE TFs and prognostic DE RBPs. The screening criteria were set as | Pearson coefficient| > 0.4 and *p* < 0.001.

### Construction of a Prognosis Signature Based on RBPs

**FIGURE 4:**
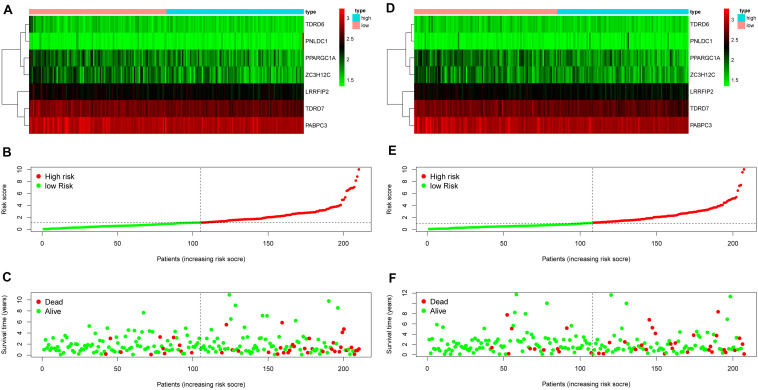
Construction of an RBP-related risk signature and validation of the prognostic value in the testing set. **(A)** Heatmap of the seven-RBP expression profiles in the high- and low-risk groups for the training set. **(B)** The seven RBPs-based risk score distribution in the training set. **(C)** Distribution of the risk score based on the seven-RBP signature for patient survival status in the training set. **(D)** Heatmap of the seven-RBP expression profiles in the high- and low-risk groups for the testing set. **(E)** The seven RBPs-based risk score distribution in the testing set. **(F)** Distribution of the risk score based on the seven-RBP signature for patient survival status in the testing set.

The 13 prognostic DE RBPs were applied to construct a prognostic model based on multiple Cox regression analysis. Cross-validation was performed to minimize overfitting. A risk-score formula consisting of seven RBPs was obtained as follows: risk score = (−2.01089 × TDRD6) + (−1.06396 × TDRD7) + (−0.83990 × PPARGC1A) + (−0.99781 × PABPC3) + (−1.3002 9 × LRRFIP2) + (−0.58829 × ZC3H12C) + (0.34108 × PNLD C1). The forest plot for the seven-RBP signature was shown in Supplementary Figure 3. The risk scores of patients in the training set and the testing set were calculated with the above formula. The median risk score of the training set was used for the cutoff value, by which the training set and the testing set were divided into high- and low-risk group. The heatmap showed the expression profiles of the seven RBPs in the training group (Figure 4A). The ranked risk scores of patients in the training set are shown in Figure 4B. The survival status for each patient was plotted, respectively (Figure 4C). The expression profiles of the seven RBPs in the testing set are presented in Figure 4D. The ranked risk scores of patients and the survival status for each patient in the testing set were plotted, respectively (Figures 4E,F). The results suggested that patients in the high-risk group showed a higher mortality rate than those in low-risk group both in the training set and the testing set.

#### Validation of the RBPs-Related Risk Signature

**FIGURE 5:**
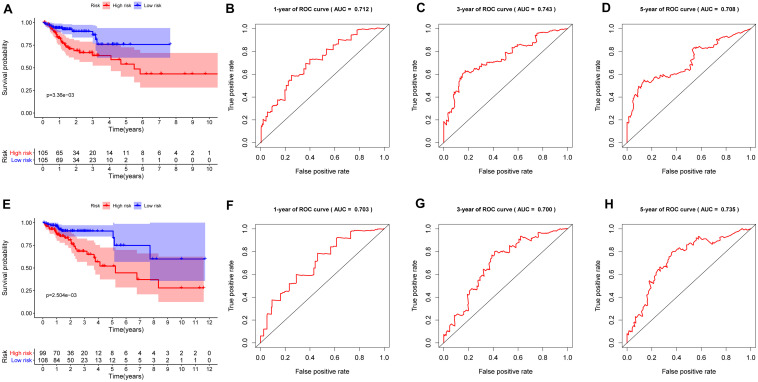
Kaplan–Meier analysis and receiver operating characteristic (ROC) curve analysis in the training set and the testing set. **(A)** Kaplan–Meier analysis of the overall survival of patients in the high- and low-risk groups in the training set based on the RBP-related risk signature. **(B)** ROC curve for 1 year in the training set. **(C)** ROC curve for 3 years in the training set. **(D)** ROC curve for 5 years in the training set. **(E)** Kaplan–Meier analysis of the overall survival of patients in the high- and low-risk groups in the testing set based on the RBP-related risk signature. **(F)** ROC curve for 1 year in the testing set. **(G)** ROC curve for 3 years in the testing set. **(H)** ROC curve for 5 years in the testing set.

To investigate the prognostic value of the RBP-related risk signature, Kaplan–Meier survival analysis was performed between high- and low-risk groups in the training set and the testing set. The high-risk group had a significantly poorer OS than that of the low-risk group both in the training set (Figure 5A) and the testing set (Figure 5E). In addition, the ROC analysis was performed to evaluate the predictive efficiency of the RBP-related prognostic signature. The areas under the ROC curve (AUCs) for the risk signature on OS at 1, 3, and 5 years were 0.712, 0.743, and 0.708 in the training set (Figures 5B–D). The AUCs for the risk signature on OS at 1, 3, and 5 years were 0.703, 0.700, and 0.735 in the testing set (Figures 5F–H). The Kaplan–Meier analysis and the ROC analysis showed that the risk signature could predict the survival status of patients with high accuracy.

### The RBP-Related Prognosis Signature Is an Independent Prognostic Index

To assess whether the prognosis signature we constructed is independent of other clinical factors, a univariate Cox proportional hazards regression and a multivariate Cox regression analysis were performed on the seven-gene signature together with gender, age, stage. Univariate Cox analysis result showed that RBP-related prognosis signature [hazard ratio (HR) = 1.332, 95% confidence interval (CI) = 1.211–1.465, *p* < 0.001] was significantly correlated with the survival of patients in the training set (Table 2). After multivariable adjustment by other clinical factors, the prognosis signature (HR = 1.252, 95% CI = 1.136–1.380, *p* < 0.001) was still significantly associated with survival of patients (Table 2), which suggested that the RBP-related prognosis signature is an independent prognostic factor associated with survival of patients with CRC.

**TABLE 2:** Univariate and multivariate Cox proportional hazards regression analysis of the RBP-related signature and clinical factors with overall survival in the training set.

Variables	Univariate analysis			Multivariate analysis		
	HR	95% CI	*p*-Value	HR	95% CI	*p*-Value
Gender	1.315	0.701–2.466	0.394	1.050	0.537–2.053	0.887
Age	1.034	1.006–1.063	**0.016**	1.038	1.010–1.067	**0.007**
Stage	2.422	1.656–3.541	**<0.001**	2.486	1.659–3.723	**<0.001**
Risk score	1.332	1.211–1.465	**<0.001**	1.252	1.136–1.380	**<0.001**

*Bold values significantly changed (*p* < 0.05).*

### Construction of a Nomogram Based on the Prognostic Signature in the Training Set

**FIGURE 6:**
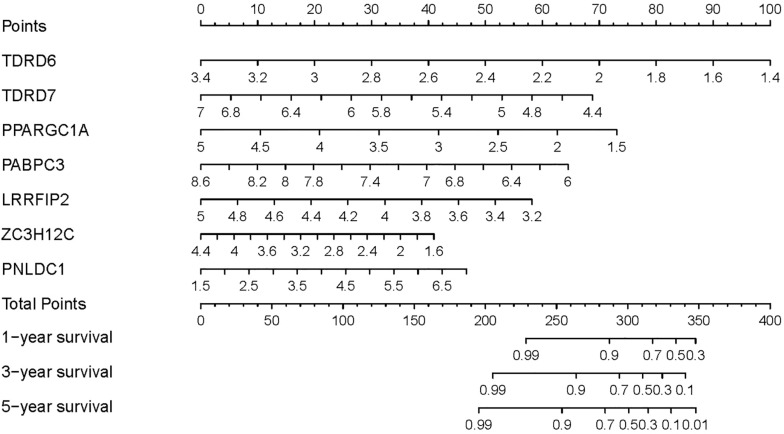
Prognostic nomogram based on the RBP-related signature for prediction of 1-, 3-, and 5-year survival rates.

To provide a quantitative method to predict the survival probability of CRC patients, a prognostic nomogram was established based on the seven RBPs of the prognostic signature using Cox proportional hazards regression analysis to predict 1-, 3-, and 5-year OS of CRC patients (Figure 6). To calculate the probability of survival at a certain point in time, the patient’s total score can be obtained by adding the scores corresponding to the values of each predictive variable and then reading the corresponding risk or probability of survival from the total score.

## Discussion

RNA-binding proteins play a vital role in the development and progression of carcinogenesis with various mechanisms. However, only a small number of RBPs have been studied in depth to confirm their role in tumor progression. Until now, there is no systematic study on RBPs in CRC. In the present study, we identified 177 DE RBPs between CRC tissues and normal tissues from TCGA dataset. To explore the potential functions of the DE RBPs, an integrated analysis was performed. We analyzed signaling pathways these DE RBPs involved in, constructed PPI network, and regulatory network between prognostic DE RBPs and TFs, which would provide a comprehensive understanding of the functions of RBPs in CRC. To further explore the clinical significance, we constructed an RBP-related prognostic model to predict the OS of CRC patients. These results provide novel biomarkers for prognosis of CRC patients.

For GO enrichment analysis, the BP of DE RBPs is mainly enriched in ncRNA metabolic process, RNA phosphodiester bond hydrolysis, ncRNA processing, nucleic acid phosphodiester bond hydrolysis, RNA localization, ribosome biogenesis, and so on. MicroRNAs, long non-coding RNAs, and small interfering RNA all belong to ncRNA. In recent years, it has become increasingly apparent that ncRNAs play a vital role in human cancer (Esquela-Kerscher and Slack, 2006; Hammond, 2007; Croce, 2009; Gibb et al., 2011). The hyperactivation of ribosome biogenesis, which can be caused by oncogenes or the loss of tumor suppressor genes, plays a vital role in cancer initiation and progression (Orsolic et al., 2016; Truitt and Ruggero, 2017). The upregulation of ribosome biogenesis during G1/S arrest can promote epithelial-to-mesenchymal transition, which is associated with tumor development and metastasis (Prakash et al., 2019). Drugs inhibiting ribosome biogenesis may offer an effective treatment for cancer (Bruno et al., 2017). For KEGG pathway enrichment, DE RBPs are mainly involved in mRNA surveillance pathway, ribosome biogenesis in eukaryotes, RNA transport, and RNA degradation. The mRNA surveillance pathway is a quality control mechanism that detects and degrades abnormal mRNAs (Moore, 2005). Non–sense-mediated mRNA decay (NMD) is one of the most common mRNA surveillance, which is involved in the detection and decay of mRNAs containing premature termination codons (Hentze and Kulozik, 1999). Inhibition of the oncogenic activity of NMD, producing several encoded mutant proteins with deleterious activity, may be an effective strategy for the personalized treatment of microsatellite instability CRC (Bokhari et al., 2018). Tumor suppressor genes are prone to NMD-induced nonsense mutations, which were reported in a series of tumors, including stomach cancer, ovarian cancer, ovarian cancer, breast cancer, and kidney cancer (Popp and Maquat, 2018). RBPs are crucial components in RNA metabolism, resulting in highly organized subcellular localization, mRNA translation, and RNA degradation and then exert regulatory effects on cancer progression (Saunus et al., 2008; Shen and Pili, 2008). These results suggest that DE RBPs may be involved in CRC progression by regulating multiple BPs, such as NRA metabolism, RNA processing, ribosome biogenesis, and mRNA surveillance.

To further explore the potential functions of DE RBP in CRC, PPI network was constructed, and the critical module was screened. Some RBPs among the critical module have been reported to be involved in cancer progression. RRS1, a nuclear protein involved in ribosome biogenesis, has been reported to regulate cancer progression in hepatocellular carcinomas (HCCs) and breast cancer. RRS1 plays a critical role in cell proliferation, colony formation, cell apoptosis, and cell cycle distribution in human HCC cells (Wang et al., 2017). In breast cancer, both the copy number and the mRNA expression of RRS1 increased in cancer tissues compared with normal tissues, and RRS1 overexpression was significantly correlated with lymph node metastasis and poor survival (Song et al., 2018). RRP12 was crucial for the survival of osteosarcoma cell line U2OS during cytotoxic stress via the repression of p53 stability, suggesting that target RRP12 may enhance the chemotherapeutic effect in cancers (Choi et al., 2016). Although some of the RBPs among the crucial modules have been linked to cancer development or resistance to cytotoxic stress, their mRNA targets and mechanism of action are still unclear. In-depth functional research on the RBPs among the critical modules will provide a new way to explore the pathogenesis of CRC, as well as the drug-sensitive targets for CRC treatment. Genes associated with prognosis usually get more attention. In the present study, 13 prognostic DE RBPs were identified by univariate Cox proportional hazards regression analysis (Table 1). Some of the identified prognostic DE RBPs have been reported associated with tumor progression, such as RBM47, LUZP4, AFF3, PPARGC1A, PPARGC1B, PABPC3, and PNLDC1. RBM47 has been identified as a suppressor of breast cancer progression and metastasis, and patients with a low level of RBM47 tended to have a poor clinical outcome (Vanharanta et al., 2014), which suppresses the metastasis of breast cancer by stabilizing transcripts of Dickkopf Wnt signaling pathway inhibitor (Vanharanta et al., 2014). Reduced levels of RBM47 have also been observed in non–small cell lung cancer (NSCLC) patients, which correspond to a poorer prognosis and more advanced disease (Shen et al., 2020). RBM47 disrupted NSCLC progression through stabilizing AXIN1 mRNA and consequently suppressing Wnt/β-catenin signaling (Shen et al., 2020). Other mechanisms of RBM47-mediated tumor inhibition in lung cancer have also been reported, RBM47 plays a tumor-suppressive role in lung cancer through inhibiting Nrf2 activity (Sakurai et al., 2016). Downregulation of RBM47 in CRC may promote epithelial–mesenchymal transition and metastasis (Rokavec et al., 2017). RBM47 positively regulates the expression of p53 at the transcriptional level and controls the expression of p21 indirectly through regulation of the p53 promoter activity (Radine et al., 2020). Our analysis also showed RBM47 was downregulated in CRC tissues (log_2_FC = -1.59, adjusted *p* = 3.92 × 10^–21^; Supplementary Table 1), and low level of RBM47 corresponding to a worse prognosis (HR = 0.549608, *p* = 0.043253; Table 1). LUZP4, also called CT-8 or HOM-TES-85, is an mRNA export adaptor required for melanoma proliferation (Viphakone et al., 2015). LUZP4 is upregulated in a range of cancers, including lung cancer, ovarian cancer, and glioma (Türeci et al., 2002). Consistent with these results, the expression of LUZP4 in CRC was also significantly higher than that in normal tissues (log_2_FC = 3.28, adjusted *p* = 5.60 × 10^–13^; Supplementary Table 1). Univariate Cox proportional hazards regression analysis suggested an increased risk for CRC patients while the LUZP4 level was high (HR = 9.613842, *p* = 0.001738; Table 1). AFF3 is a new target of Wnt/β-catenin pathway involved in adrenocortical cancer, acting on transcription and RNA splicing (Lefèvre et al., 2015). Overexpression of AFF3 in breast cancer was associated with tamoxifen resistance and worse OS (Shi et al., 2018). However, downregulation of AFF3 was observed in CRC tissues (log_2_FC = −2.92, adjusted *p* = 2.54 × 10^–22^; Supplementary Table 1). AFF3 may have different roles in cancers. PPARGC1A is upregulated and facilitates lung cancer metastasis (Li et al., 2017). Genetic variants in PPARGC1A and PPARGC1B have been reported in breast tumors and were associated with familial breast cancer susceptibility (Wirtenberger et al., 2006). PABPC3 has been reported to be a breast cancer candidate gene that may be associated with breast cancer (Kim et al., 2017). PNLDC1 mRNA was altered in several tumor types by analyzing more than 6,000 adult and pediatric tumors (Saghafinia et al., 2018). Although these prognostic DE RBPs were documented to be involved in tumor development, the functions and mechanisms of the remaining DE RBPs other than RBM47 have not been fully elucidated. Some identified prognostic DE RBPs have not been associated with cancers, such as CELF4, POP1, TDRD6, TDRD7, LRRFIP2, and ZC3H12C. Even so, LRRFIP2 was identified as a component of the Wnt signaling pathway that mediates or modulates Wnt signaling through interactions with Dvl (Liu et al., 2005). LRRFIP2 may be involved in cancer progression through regulating Wnt signaling pathway.

The functions of some identified prognostic DE RBPs have not been reported. We constructed a regulatory network based on differentially expressed TFs and prognostic DE RBPs by calculating the Pearson correlation, which would contribute to a systematic understanding of prognostic DE RBPs in CRC. As shown in Figure 3E, three RBPs (TDRD7, ZC3H12C, and RBM47) may be regulated by KLF4. KLF4 is an evolutionally conserved zinc finger–containing transcription factor that regulates diverse cellular processes such as cell growth, proliferation, and differentiation (Bieker, 2001; Ghaleb et al., 2005). KLF4 displays a tumor-suppressive function, which is downregulated in CRC (Shie et al., 2000b). KLF4 overexpression in CRC cell line reduced transformation, migration, invasion, and tumorigenicity (Dang et al., 2003). Constitutive expression of KLF4 led to the inhibition of DNA synthesis and regulates the cell cycle by blocking G1/S progression (Chen et al., 2001). KLF4 suppresses CRC proliferation through upregulating p21^WAF1/Cip1^ and downregulating cyclin D1 (Shie et al., 2000a). In the present study, KLF4 expression is downregulated in CRC tissues (log_2_FC = −2.52, adjusted *p* = 2.13 × 10^–22^; Supplementary Table 2), which is consistent with previous report. The results suggest that KLF4 may exert a suppression effect through other mechanisms, such as regulating TDRD7, ZC3H12C, and RBM47. We also demonstrated that KAT2B, a histone acetyltransferase to promote transcriptional activation (Ogryzko et al., 1996), may regulate three RBPs (TDRD6, ZC3H12C, and RBM47). Moreover, KAT2B acetylates non-histone proteins (Choudhary et al., 2009; Downey and Baetz, 2016), which has been reported to be a critical regulator of p53-dependent p21 expression in response to multiple p53-activating stresses (Love et al., 2012). KAT2B also plays a role in the development and progression of cancer (Bondy-Chorney et al., 2019). Thus, KAT2B may be involved in CRC progression through regulating TDRD6, ZC3H12C, and RBM47. Although the functions of TDRD6, TDRD7, and POP1 are not clear, the regulatory network will guide further study of the functions of these RBPs. TDRD6 may be regulated by NR3C1, OGT, MAF, CBFB, POLR3G, and KATZB (Figure 3E). TDRD7 may be regulated by KLF4 and CHD7 (Figure 3E). POP1 may be regulated by CHD7, E2F1, MYC, BRCA1, and PRKDC (Figure 3E). Besides, multiple Cox regression analysis was performed to construct a risk signature for predicting survival of CRC patients, including TDRD6, TDRD7, PPARGC1A, PABPC6, LRRFIP2, ZC3H12C, and PNLDC1. Kaplan–Meier survival analysis and ROC analysis were performed in the training set and testing set to evaluate the predictive efficiency of the RBP-related prognostic signature. Results showed that the signature can predict the survival status of patients in TCGA dataset with high accuracy. Subsequently, a nomogram was built based on the RBP-related prognostic signature to predict 1-, 3-, and 5-year OS more intuitively. In summary, we investigated the expression, prognostic values, and potential functions of DE RBPs through comprehensive bioinformatics analysis. Thirteen prognostic RBPs were identified. An RBP-related prognostic model was developed that could act as an independent prognostic signature for CRC. These findings would provide new therapeutic targets and prognostic markers for CRC.

## Data Availability Statement

The raw data supporting the conclusions of this article will be made available by the authors, without undue reservation.

## Ethics Statement

This study was approved by the Ethical Committee of Central South University (China).

## Author Contributions

SX and KF conceived the concept, instructed data analysis, and revised the manuscript. YW conducted most of the data analysis prepared figures and table, and wrote manuscript draft. YC helped with some analysis and interpretation of data. KF reviewed the manuscript with input from all authors. All authors contributed to the article and approved the submitted version.

## Conflict of Interest

The authors declare that the research was conducted in the absence of any commercial or financial relationships that could be construed as a potential conflict of interest.

## Footnotes

1 https://string-db.org

2 http://cistrome.org/

## Abbreviations

AUC: area under the ROC curve
BP: biological processes
CCs: cellular compartments
CRC: colorectal cancer
DE RBPs: differentially expressed RBPs
FC: fold change
GO: Gene Ontology
HR: hazard ratio
KEGG: Kyoto Encyclopedia of Genes and Genomes
MFs: molecular functions
NSCLC: non–small cell lung cancer
OS: overall survival
PPI: protein–protein interaction
RBPs: RNA-binding proteins
ROC: receiver operating characteristic
TCGA: The Cancer Genome Atlas
TFs: transcription factors.
