# Supplementary material for: Integrated Analysis of the Functions and Prognostic Values of RNA-Binding Proteins in Colorectal Cancer
Source: Front Cell Dev Biol. 2020 Nov 5;8:595605. doi: 10.3389/fcell.2020.595605 (PMC7674310; doi:10.3389/fcell.2020.595605)
Supplement: Supplementary file 3 [file Data_Sheet_1.docx]

Supplementary Material

##
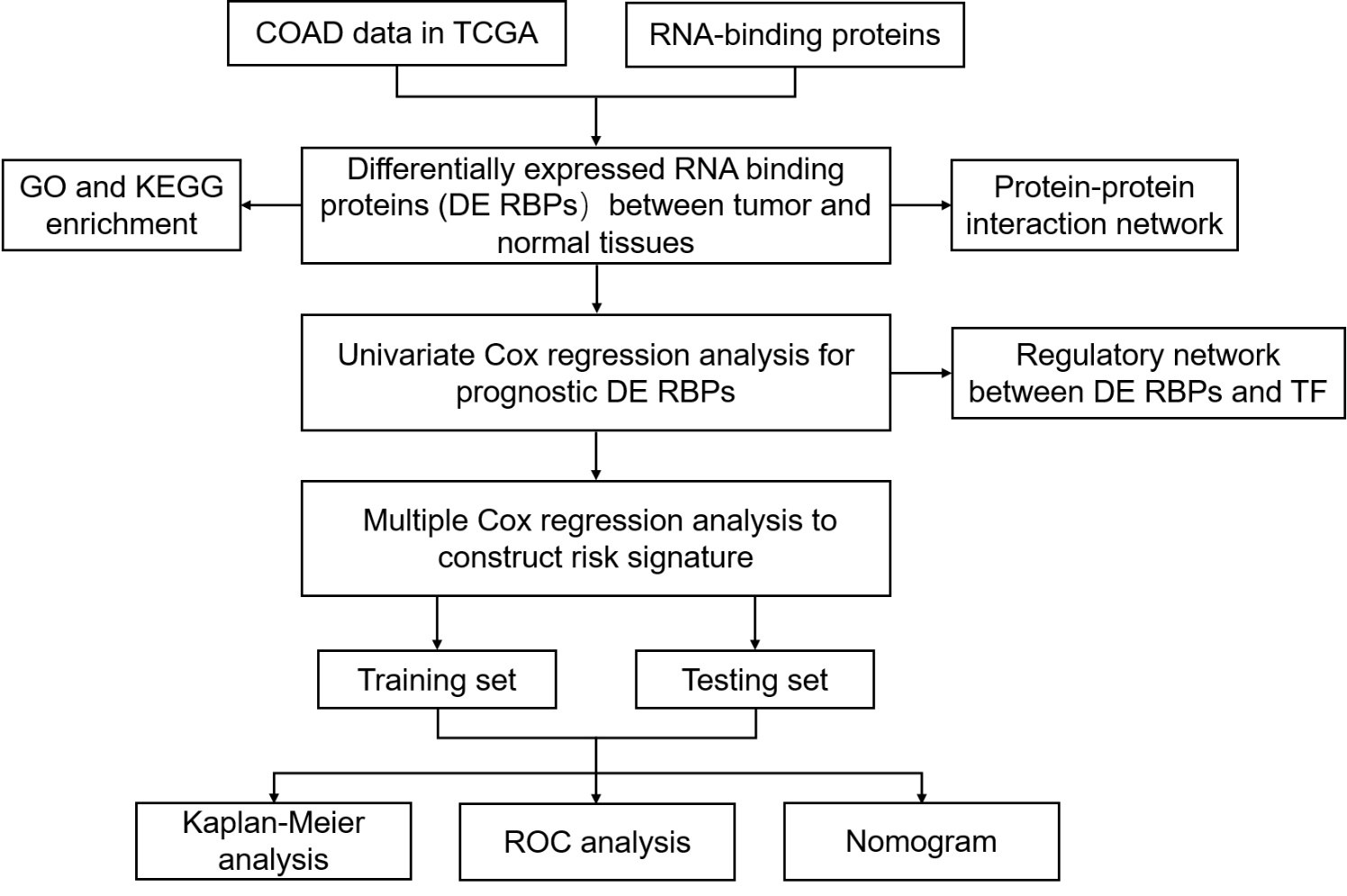
Supplementary Figures

**Supplementary Figure 1.** The workflow for analyzing the RBPs in CRC.


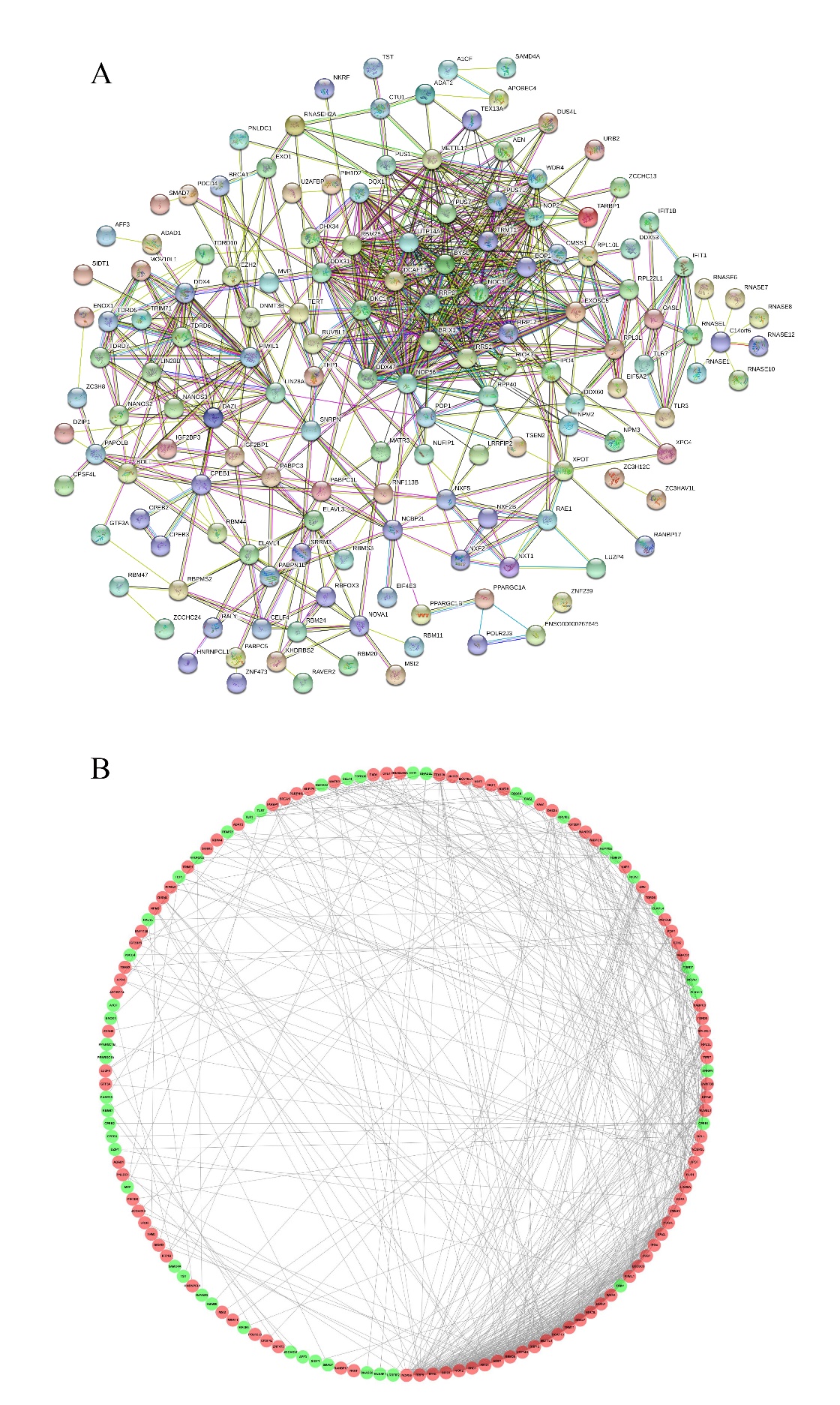
**Supplementary Figure 2.** PPI network of differentially expressed RBPs. (A) PPI network from STRING database. (B) PPI network visualized by “cytoscape”. Green circles represent down-regulated RBPs, and red circles represent up-regulated RBPs.

**
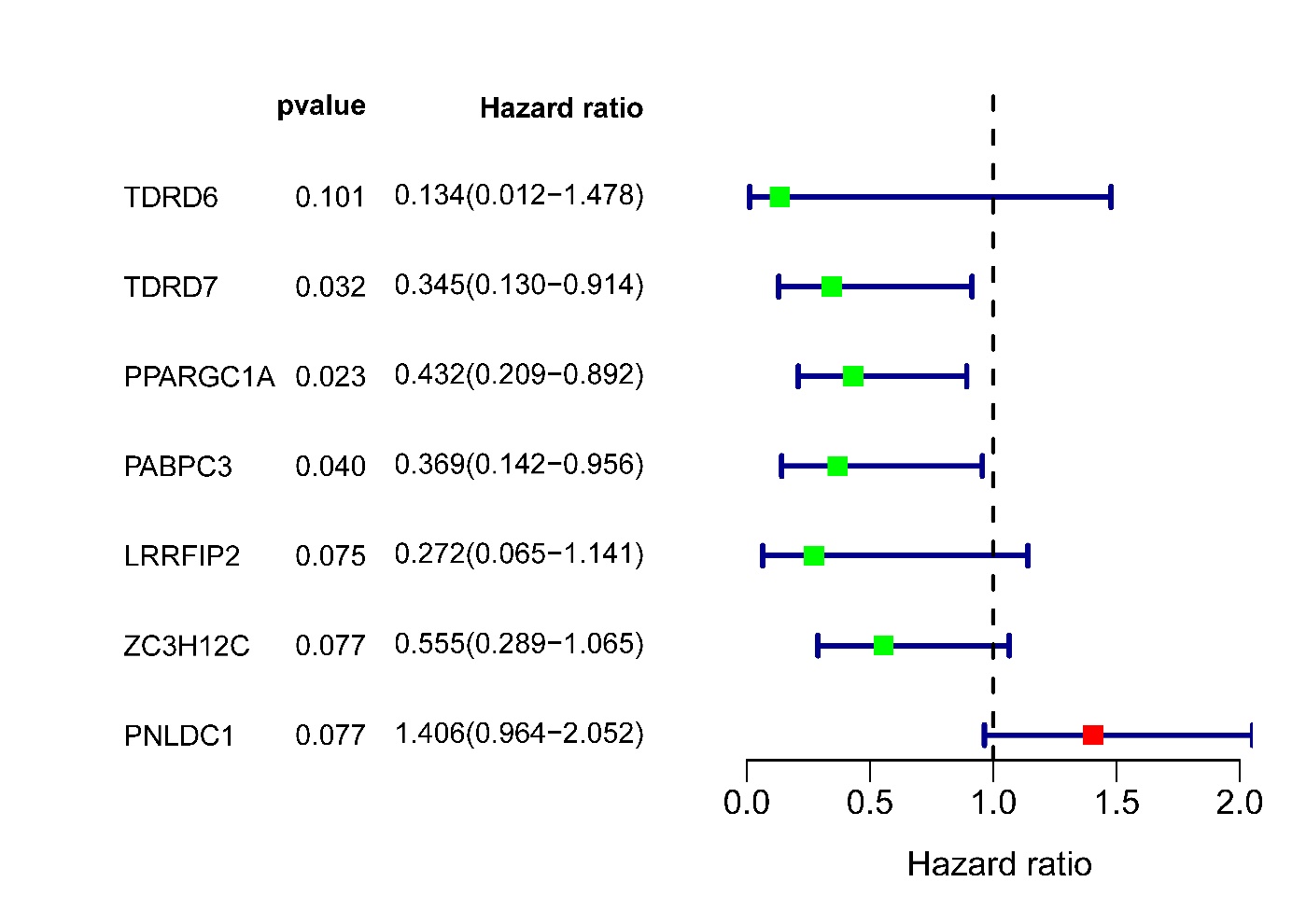
**

**Supplementary Figure 3.** Forest plot illustrating the multivariate Cox model results of each gene in the seven-RBP signature. HR and 95% CI were visually represented by the squares and error bars.

# Supplementary Tables

**Supplementary Table 1.** DE RBPs in CRC patients.

**Supplementary Table 2.** 77 differentially expressed TFs were obtained with a threshold that adjusted *p* <0.05 and |log_2_FC| ≥1.
